# Urban Tree Effects on Soil Organic Carbon

**DOI:** 10.1371/journal.pone.0101872

**Published:** 2014-07-08

**Authors:** Jill L. Edmondson, Odhran S. O'Sullivan, Richard Inger, Jonathan Potter, Nicola McHugh, Kevin J. Gaston, Jonathan R. Leake

**Affiliations:** 1 Department of Animal and Plant Sciences, University of Sheffield, Sheffield, United Kingdom; 2 Environment and Sustainability Institute, University of Exeter, Penryn, Cornwall, United Kingdom; DOE Pacific Northwest National Laboratory, United States of America

## Abstract

Urban trees sequester carbon into biomass and provide many ecosystem service benefits aboveground leading to worldwide tree planting schemes. Since soils hold ∼75% of ecosystem organic carbon, understanding the effect of urban trees on soil organic carbon (SOC) and soil properties that underpin belowground ecosystem services is vital. We use an observational study to investigate effects of three important tree genera and mixed-species woodlands on soil properties (to 1 m depth) compared to adjacent urban grasslands. Aboveground biomass and belowground ecosystem service provision by urban trees are found not to be directly coupled. Indeed, SOC enhancement relative to urban grasslands is genus-specific being highest under *Fraxinus excelsior* and *Acer* spp., but similar to grasslands under *Quercus robur* and mixed woodland. Tree cover type does not influence soil bulk density or C∶N ratio, properties which indicate the ability of soils to provide regulating ecosystem services such as nutrient cycling and flood mitigation. The trends observed in this study suggest that genus selection is important to maximise long-term SOC storage under urban trees, but emerging threats from genus-specific pathogens must also be considered.

## Introduction

Urban ecosystems provide vital goods and services to the inhabitants of cities and towns [1]. Urban trees are especially important in providing a diverse range of ecosystem services. These include organic carbon storage [2]; flood mitigation and improved water quality [3]; filtration of atmospheric pollutants, especially removing health-damaging fine particulates such as PM10 [4], [5], and particulate-bound carcinogenic polycyclic aromatic hydrocarbons [6]; absorption of toxic gasses including O_3_, SO_2_ and NOX [7]; traffic noise pollution reduction [8]; and, amelioration of the urban heat island effect [9]–[11]. In addition, urban trees and greenspaces provide wildlife habitat and aesthetic values that further contribute benefits to human wellbeing, quality of life and health [12]–[14]. Trees are a ubiquitous part of cities and towns, and have been estimated to cover between 10–67% of urban and community areas in the USA [3] and 20% of Greater London, UK [5].

The recognized importance of trees for ecosystem service provision has stimulated global efforts to increase tree cover in urban areas, for example ‘*The Big Tree Plant*’ in England run by DEFRA and the Forestry Commission [15], and the private-public run ‘*MillionTreesNYC initiative*’ in New York, USA [16]. However, these initiatives have been conducted without a clear understanding of the effects of urban trees on provision of ecosystem services belowground. Given that approximately 75% of ecosystem carbon storage occurs in soils [17], [18], the net effect of trees on soil organic carbon (SOC) stores is particularly important since, in addition to providing a sink for atmospheric carbon dioxide fixed by photosynthesis, SOC is positively associated with regulating and supporting ecosystem services such as storm-water infiltration and nutrient holding capacity [19]. Without an assessment of the impact of tree planting on SOC and other soil properties that deliver ecosystem service benefits in an urban context it is not possible fully to understand the implications of this widespread management practice.

Trees can influence the biological, chemical and physical properties of soils directly through their deep roots and litter quality and quantity [20]. Changes in soil properties following afforestation are varied and dependent on former land-use and species, particularly whether broadleaved angiosperms or gymnosperms like pines [21], [22]. Previous studies on arable land, which has strongly depleted SOC stocks [23], have shown afforestation typically results in increased carbon sequestration both above and belowground [22]. Furthermore, the English national SOC inventory reports greater storage under woodland (primary and afforested) than pasture [24]. The UK Countryside Survey found lower soil bulk density (BD) values beneath woodland compared to improved grasslands [25], indicating a greater capacity of woodland soils to absorb sustained heavy rainfall and reduce run-off and flooding. As these countrywide survey habitats will include agricultural grasslands that have been ploughed and reseeded, they include soils that have previously been depleted in SOC and experienced increased BD prior to tree planting, as well as ancient woodlands.

Recent studies have shown that urban soils hold much higher concentrations of SOC than typical arable fields, into the same range as semi-natural grasslands and woodlands [26], [27]. The extent to which urban trees can increase SOC stocks over those of urban grasslands is currently unknown, as is the relative importance of different major urban tree genera. This knowledge-gap is strategically important with the rising pan-European threats to keystone urban tree species from virulent pests and diseases, including Ash dieback, Oak decline and Oak Processionary Moth [28]–[30]. Here, we use an observational study in urban parks to examine differences in soil properties (SOC, C∶N ratio, and BD) beneath three tree genera (*Acer* spp., *Fraxinus excelsior*, and *Quercus robur*) selected for their abundance and capacity to grow into large specimens, and mixed urban woodlands in comparison to adjacent urban grassland soil. We test the hypothesis that SOC stocks and C∶N ratios would be increased in soil under trees compared to grassland, paralleling and positively correlating with greater above-ground carbon storage, whereas soil BD would be reduced under trees compared to grassland as seen in semi-natural ecosystems [25].

## Methods

### Study area

This research focussed on Leicester, a mid-sized UK city, located in the East Midlands of England (52°38′N, 1°08W). It has a human population of 310,000 [31], and covers an area of approximately 73 km^2^. The region has a temperate climate, receiving 620 mm of precipitation each year and average annual daily minimum and maximum temperatures of 6.1°C and 13.9°C, respectively [32]. Soil types within the city are dominated by deep clays, deep loam and seasonally wet deep clays and loam, according to the National Soil Map for England and Wales produced by Cranfield University. The soil types sampled in the city were: Hanslope, Whimple, Salop, Beccles 3, Ragdale and Fladbury 1.

### Sampling strategy

A GIS was used to select randomly urban parks for soil sampling within the city of Leicester and permission was granted for the work by the Leicester City Council. The land-use history of each park was assessed in a GIS using the series of historic Ordnance Survey maps dating back to 1887. No park had previously been built upon, one park and two country houses and grounds that went on to form parks were in existence in 1887, the remainder were agricultural land at this time. Over the following years to the present day the remainder of the parks were established as the city expanded into the surrounding agricultural landscape, with the two recent parks developed on farmland within the last 20–30 years (see Table S1 for site specific land-use history). At each park an initial assessment was made for the presence of individual trees that ranged in size from saplings to large mature specimens within our target genera, specifically isolated specimens of *Quercus robur*, *Fraxinus excelsior*, *Acer* spp. (comprised of *Acer pseudoplatanus* and *Acer platanoides*), or patches of mixed woodlands. The selected trees ranged in diameter at breast height (DBH) from 2.5 cm to 197 cm and in biomass from 1.3 kg to 61 tonnes for the largest mature *Q. robur* specimen (see Table S1). The tree genera were selected because of their importance in parks in Leicester and national abundance, *Q. robur* being the commonest tree and *F. excelsior* the second most common as an individual tree or within small patches of woodland in Great Britain [33]. *A. pseudoplatanus* is the fourth most common tree species in small patches of British woodland [33].

Where a park contained one or more of the tree genera and/or mixed woodland at least one grassland site was also identified for sampling. Each grassland site was situated in proximity to the tree sites identified, but was also over 50 m from any individual or patch of trees, to ensure that it was outside the influence of the trees. A GIS layer obtained from Leicester City Council was used to check that management at each grassland site was uniform, specifically that the regularity of mowing at all sites was approximately 25 times per year, these park grasslands were not irrigated nor did they receive any fertiliser input. The grassland sites were selected to act as a direct comparison (or paired sample) at each specific location (park) with the tree sample.

At each site, tree species, height and DBH were recorded within a 5×5 m quadrat centred on individual isolated trees within grassland or in mixed woodland. Soils were sampled in approximately 7 cm increments to 1 m depth [26], the reference depth for the national SOC inventory [21], [24], under target tree genera, mixed woodland and grasslands. Under isolated trees soil samples were taken within 1 m of the trunk to ensure that all samples were taken beneath the canopy of even the small immature trees. In total, soils were sampled beneath 12 specimens of *Quercus robur*, 11 of *Fraxinus excelsior*, 12 of *Acer* spp. In addition soils were sampled beneath mixed urban woodland at 8 sites, and urban grassland at 15 sites.

In addition to the three target tree genera specified a further six species; *Acer campestre*, *Corylus avellana*, *Crataegus monogyna*, *Salix caprea*, *Tilia* x *europaea* were identified in the mixed urban woodlands. Measured tree DBH and height were used to estimate aboveground biomass with allometric equations. For each species, where multiple equations were available they were combined to produce a generalised biomass prediction. Where species-specific equations did not exist genus level equations were used, following the methodology recently used to derive tree aboveground biomass across the city of Leicester [2]. However, allometric equations to predict biomass of urban trees specifically are scarce [34], thus those used were derived from European and North American forested ecosystems [2], [35]–[37].

### Soil sample preparation and analysis

Soil samples were analysed for SOC, C∶N ratio and BD using established procedures. In brief, soil samples were dried at 105°C for 24 hours, weighed, ball milled to homogenise, and passed through a 1 mm sieve [26]. Soil BD (g cm^−3^) was calculated after removing the dry weight of matter greater than 1 mm [38]. Soils were analysed in duplicate for total N concentration (mg g^−1^) in a CN analyser [26]. Inorganic carbon was removed from 2.5 g of soil sample by adding 10 ml 5.7 M HCl, samples were centrifuged at 1800 *g* for 10 minutes, supernatant discarded and dried at 105°C. Subsequent CN analysis in duplicate determined SOC concentration (mg g^−1^) [26].

### Statistical analysis

All dependant variables (SOC Concentration, SOC Density, Soil C∶N & Soil Bulk density) were checked for normality and homoscedasticity, and log transformed where necessary. Each of these dependant variables was analysed using general linear mixed effects models. The maximal model included vegetation cover as a 5 level fixed factor (urban grassland, *Q. robur, F. excelsior, Acer* spp. and mixed woodland). Soil depth (at which the sample was taken) and the biomass of the individual tree in proximity to the soil sample or in mixed woodland the biomass of all trees within the 5×5 m quadrat were incorporated as covariates in the model, and urban park identity was included as a random (intercept) factor to account for differences in length of time since park establishment and geographic location within the city. Initial models included tree biomass as a variable, however this had no predictive power (as the confidence intervals spanned zero) for any measured soil property and therefore it was removed as a variable from all subsequent models. In all cases model simplification was attempted by comparing all possible simpler model subsets using Akaike's information criterion (AIC). The maximal model, containing all the predictor variables, was always found to be the top model (based on AIC) and used in the subsequent inferences. Pseudo R^2^ values were calculated using the methods of Nagelkerke [39]. Mixed models were carried out using package lme4. F and p values were calculated using Satterthwaite approximations [40] to determine denominator degrees of freedom in package lmerTest. All tests were carried out in the R language and environment [41]. Raw data are available in Table S1.

## Results

**Figure 1:**
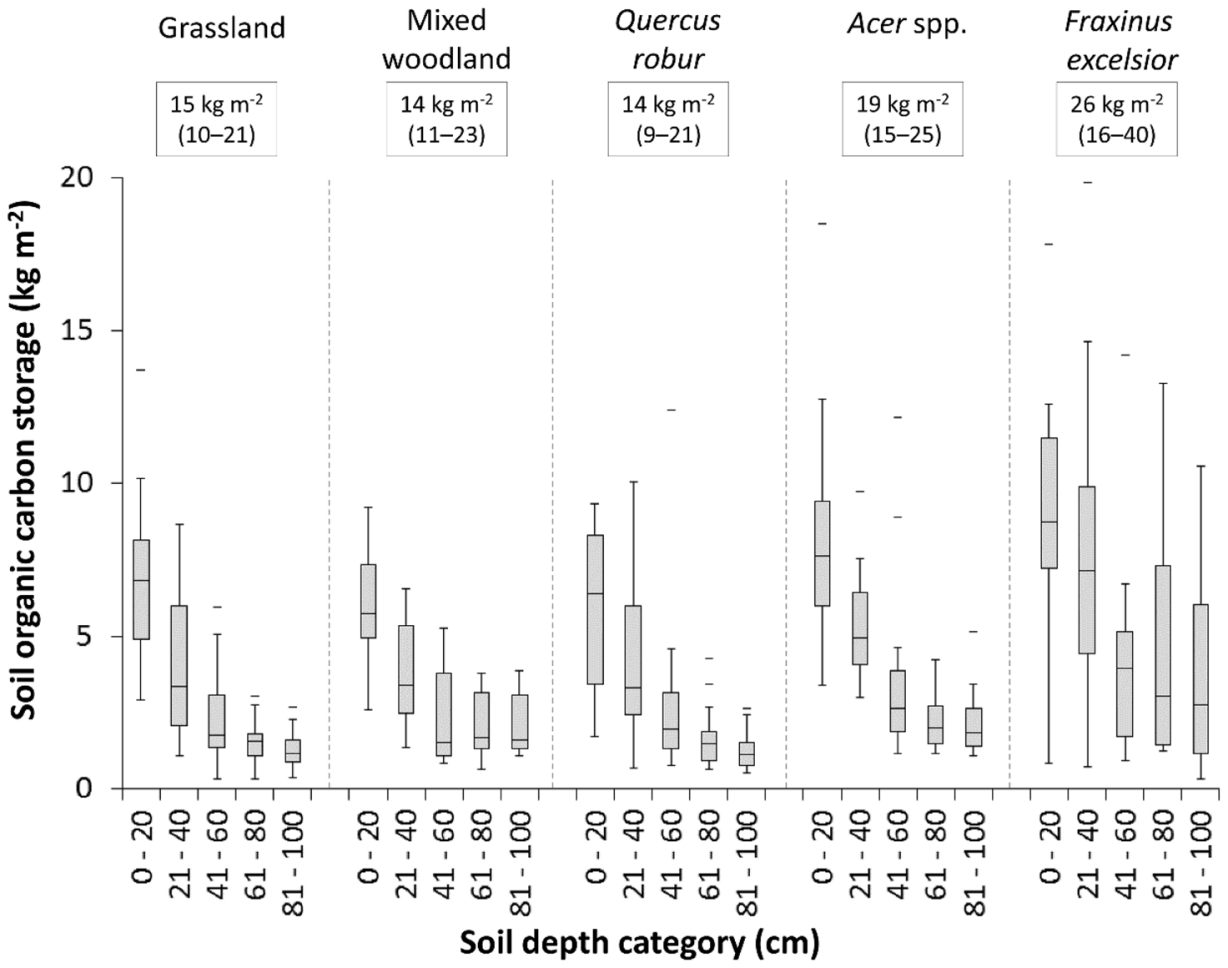
Soil organic carbon storage within each 20(summed median values are displayed in text boxes, values in parenthesis are total 25^th^ and 75^th^ percentiles), beneath *Quercus robur* (n = 12), *Fraxinus excelsior* (n = 11), *Acer* spp. (n = 12), mixed woodland (n = 8) and grassland (n = 15) by depth class. The horizontal line within the box indicates median, box boundaries indicate 25^th^ and 75^th^ percentiles, whiskers indicate highest and lowest values, horizontal lines above or below whiskers indicate outliers.

**Figure 2:**
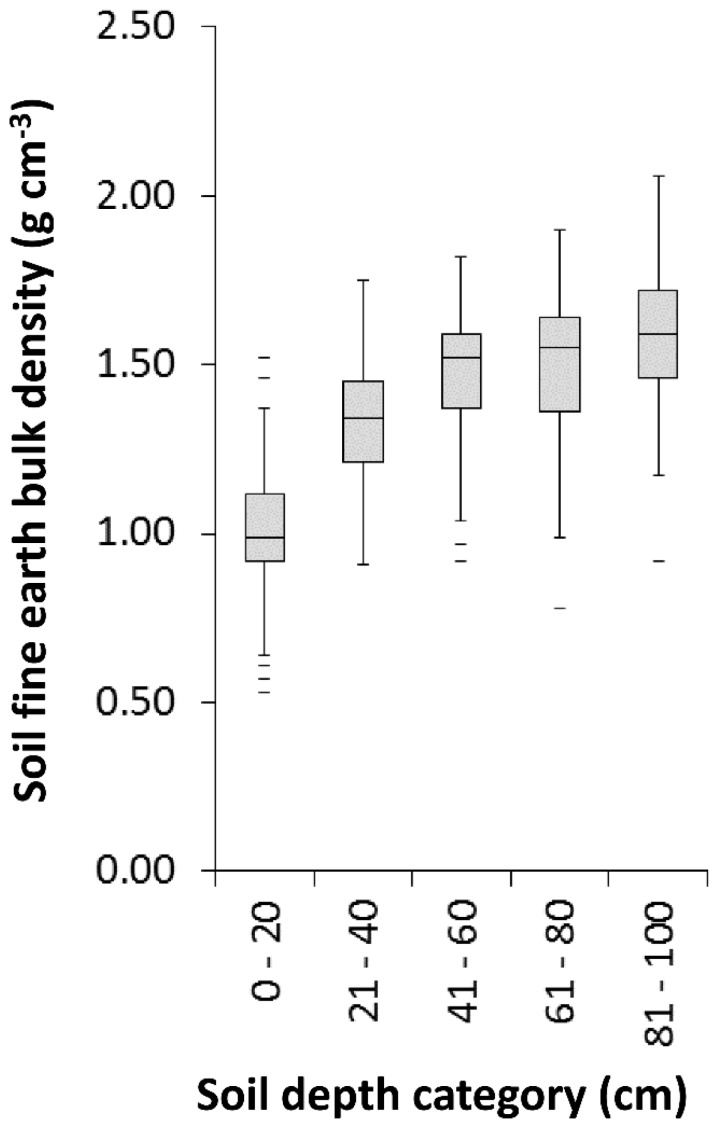
Soil bulk density in each 20 cm depth category. The horizontal line within the box indicates median, box boundaries indicate 25^th^ and 75^th^ percentiles, whiskers indicate highest and lowest values, horizontal lines above or below whiskers indicate outliers.

There were significant differences in SOC concentration and amount per soil volume (SOC density) between the three tree genera, mixed urban woodland and grassland (*F* = 9.95, *p*<0.001; and *F* = 12.51, *p*<0.001, respectively). Soil depth had a strong effect on SOC concentration (*F* = 609.89, *p*<0.001) and density (*F* = 456.27, *p*<0.001). Median SOC concentration and density were greatest beneath *F. excelsior* throughout the depth profile, followed by *Acer* spp., with no difference between *Q. robur*, mixed woodland, and grassland (Fig. 1). Model pseudo R^2^, including both soil depth and tree cover, explained 59% and 57% of variation in SOC concentration and density respectively. Estimates of total SOC storage, based on summed median values for each depth category, ranged from 14–26 kg SOC m^−2^ with lowest storage beneath *Q. robur*, mixed woodland, and grassland, and the highest under *F. excelsior* (Fig. 1). The explanatory power of the mixed effects model for C∶N ratio was low, with a pseudo R^2^ explaining only 11% of the variation in the data (soil depth *F* = 20.74, *p*<0.001; vegetation cover *F* = 7.579, *p*<0.001). The effect of trees on BD was not significant (*F* = 1.24, *p* = 0.295). Soil depth was the most important predictor of BD (F = 244.35, p<0.001, model pseudo R^2^ = 49%), with increased median BD with depth from 0.99–1.59 g cm^−3^ between 0–20 cm to 80–100 cm (Fig. 2). The mixed effects models including tree biomass as a variable (excluding grassland as this cover type has no woody biomass) revealed that the effect size of tree biomass on SOC, C∶N ratio and BD was small, and as confidence intervals spanned zero had no predictive power (see Table S2 for model statistics).

## Discussion

Previous research in a typical UK city, Leicester, has shown that 97% of carbon present in aboveground ecosystem biomass is found in trees, with average storage increasing from 0.2 kg m^−2^ in herbaceous vegetation (most commonly urban grassland) to 28.5 kg m^−2^ in trees [2], affirming the importance of urban trees in aboveground carbon sequestration. In contrast we found that differences in SOC under trees compared to grassland were more modest and genus-specific and, surprisingly, did not occur under mixed woodland or *Q. robur*, even though several specimens of the latter had a trunk diameter of over 1.5 m. No significant differences in SOC concentration beneath urban forests and grasslands were found in Baltimore, USA [42], corresponding with our findings for mixed woodlands. Nonetheless, we did find increased SOC storage under *F. excelsior* and, to a lesser extent, *Acer* spp., with gains of 11 kg m^−2^ and 5 kg m^−2^ respectively compared to the adjacent grasslands (Fig. 1).

The effects of *F. excelsior* are of particular interest in light of current concerns about the impact of ash die-back disease on this widespread and abundant species in Europe and the UK, where it is the second most abundant tree in small woodland patches and the second most common individual tree in the countryside [33]. *A. pseudoplatanus* and *F. excelsior* are mesophilic species that thrive on well-watered alluvial soils, but the former is more drought susceptible [43]. The clear SOC enrichment throughout the soil profile under *F. excelsior*, especially from 40–100 cm depth, could be attributed to several factors. This species produces a greater root mass than *A. pseudoplatanus* and *Q. robur*, and establishes its extensive deep root network more quickly than other broadleaved species [44], and is especially well adapted to clay-rich soils such as those found in Leicester. Organic carbon introduced by tree roots into clay rich subsoils will experience long residence times [45].

Compared to park grassland, none of the tree genera or mixed woodland altered soil BD, which is consistent with previous reports of the absence of effects of broadleaved trees planted in pasture [46]. However, the urban trees could still aid storm-water drainage along root channels [20]. Similarly, soil C∶N ratio, one of the major controls of N availability and leaching [47], was also unaffected by trees.

These data highlight the often overlooked importance of urban park grasslands as contributors to belowground ecosystem service provision, particularly SOC storage, which we show exceeds typical values for agricultural grassland by 23% [24]. Urban park management may reduce litter inputs from trees relative to grassland as mowings are not collected but autumn leaves are removed from beneath individual trees. However, it remains unclear how important these management practices are as leaves were not removed from beneath the stands of mixed woodland we sampled, yet these showed no significant increase in SOC storage compared to adjacent grassland. Carbon inputs into the soils under grassland and park trees are likely to be strongly influenced by roots. Mean residence time of root carbon in soil is typically 2.4 times that of shoot carbon, due to higher concentrations of the more recalcitrant components such as lignin, so that SOC is often mainly derived from root inputs [48].

This observational study aimed to provide a first indication of the long-term effect of trees in urban areas on soil properties. As our approach was non-experimental we cannot be certain whether the sampled trees were planted or naturally regenerated from seed, and we have to assume that any differences in soil conditions under trees and adjacent grassland are due to the trees. However, our strategy of sampling soil under trees of very different sizes enabled us to investigate if there were any relationships between tree size (as a proxy for tree age) and soil properties across a range of urban parks. Indeed, given the range of tree sizes we studied, the trees will likely have ranged in age from a decade to several centuries- a timespan difficult to achieve in experimental manipulation studies. Perhaps one of the most surprising findings arising from this was the absence of clear effects of tree size on soil carbon storage, especially for oaks where the largest individuals were 1.6 m–2.0 m DBH.

Tree planting within urban areas is one of a range of environmental management techniques used which impact, either intentionally or unintentionally, on ecosystem service provision [1]. Although tree planting is known to increase ecosystem service provision aboveground [2]–[14], we now show for the first time for urban trees that this benefit does not consistently extend into the soil system as, contrary to our original hypotheses, there is no direct relationship between aboveground tree biomass and SOC concentration, soil C∶N ratio and soil BD.

We demonstrate the importance of targeted tree genus selection to maximise the long-term belowground ecosystem service benefits of urban tree planting with respect to SOC. Further research should seek to elucidate the belowground effects of other common tree species on a range of soil types and climate zones across urban areas globally, better to inform urban policy and planning. Our findings that *F. excelsior* makes an important contribution to enhancing urban SOC stocks coincides with the first record of ash dieback disease spreading into our study region (Leicestershire) [49]. This raises important questions about the likely future impacts of loss of this species on SOC stocks nationally. Furthermore, it highlights the importance of long-term planning in cities and towns to couple the ecosystem service benefits of tree planting with disease and climate change resilient urban tree populations in the future.

## Supporting Information

Table S1 **Raw data file.**
(XLSX)

Table S2 **Results from mixed effect model and model averaging for models including tree biomass.**
(DOCX)
