# Supplementary material for: Urban Tree Effects on Soil Organic Carbon
Source: PLoS One. 2014 Jul 8;9(7):e101872. doi: 10.1371/journal.pone.0101872 (PMC4087013; doi:10.1371/journal.pone.0101872)
Supplement: Table S2 — Results from mixed effect model and model averaging for models including tree biomass. (DOCX) [file pone.0101872.s002.docx]

**Urban tree effects on soil organic carbon**

Jill L. Edmondson, Odhran S. O’Sullivan, Richard Inger, Jonathan Potter, Nicola McHugh, Kevin J. Gaston, Jonathan R. Leake

**Table S2:** Results from mixed effect model and model averaging for models including tree biomass. Pseudo R^2^ values are calculated using the methods of Nagelkerke (1991). Z & *p* values are based on the final averaged model. Relative importance is the proportion of all the top models which contained the term for tree biomass. For comparison all top models for all variables contained soil depth in all top models (relative importance =1). Vegetation cover was retained in all the top models for all variables apart from the model for soil bulk density where the relative importance was 0.21.

| Variable | Pseudo R^2^ | z-value | *p* value | Relative Importance |
| --- | --- | --- | --- | --- |
| SOC Concentration | 0.58 | 0.802 | 0.42 | 0.33 |
| SOC Density | 0.51 | 0.61 | 0.53 | 0.54 |
| Soil C:N | 0.16 | 1.49 | 0.13 | 0.51 |
| Soil Bulk Density | 0.49 | 0.85 | 0.39 | 0.33 |

**References**

1. Nagelkerke, N.J.D. (1991) A note on a general definition of the coefficient of determination. *Biometrika*. **78**, 691-692.
